# Radiofrequency Ablation of the Atherosclerotic Plaque: a Proof of Concept Study in an Atherosclerotic Model

**DOI:** 10.1007/s12265-017-9743-3

**Published:** 2017-03-31

**Authors:** Guilielmus H.J.M. Ellenbroek, Gerardus P.J. van Hout, Saskia C.A. de Jager, Leo Timmers, Aryan Vink, Roel Goldschmeding, Petra van der Kraak, Gerard Pasterkamp, Imo E. Hoefer, Petrus A. Doevendans, Yolande Appelman

**Affiliations:** 1 Laboratory of Experimental Cardiology, University Medical Center Utrecht, Heidelberglaan 100, Internal mail no G03.550, 3508 GA Utrecht, The Netherlands; 2 Department of Cardiology, University Medical Center Utrecht, Utrecht, The Netherlands; 3 Department of Pathology, University Medical Center Utrecht, Utrecht, The Netherlands; 4 Department of Clinical Chemistry and Hematology, University Medical Center Utrecht, Utrecht, The Netherlands; 5 Netherlands Heart Institute, Utrecht, The Netherlands; 6 Department of Cardiology, VU Medical Center, Amsterdam, The Netherlands

**Keywords:** Radiofrequency ablation, Atherosclerosis, Plaque progression, Neovascularization, Vasa vasorum, Smooth muscle cell

## Abstract

Increased plaque vascularization is causatively associated with the progression of unstable atherosclerotic vessel disease. We investigated the safety and efficacy of heat-generating radiofrequency ablation (RFA) in reducing the number of vessels in the plaque and adventitia and its effect on plaque size and composition. To this end, New Zealand White rabbits were fed a cholesterol-enriched diet and subjected to balloon denudation of the infrarenal aorta to induce atherosclerotic plaque formation. After 13 weeks, the proximal or distal half of the infrarenal aorta was exposed to transluminal RFA. The untreated half served as an intra-individual control. Optical coherence tomography (OCT) was performed directly after RFA. We found that RFA on the rabbit atherosclerotic plaque is safe and leads to decreased intraplaque vessel density and smooth muscle cell content but does not affect other components of plaque composition or size.

## Introduction

Atherosclerosis is the most prevalent cause of cardiovascular disease (CVD) worldwide and the main cause of death in the Western world [1]. Progression of atherosclerosis may lead to plaque instability and give rise to an acute myocardial infarction (AMI), cerebrovascular accident (CVA), or acute peripheral vascular disease. Increased vasa vasorum density results in a higher influx of inflammatory cells and lipid deposition, culminating in unstable plaque formation [2]. In addition, immature and leaky neovessels originating from the vasa vasorum consequently increase the risk for intraplaque hemorrhage, thereby increasing plaque vulnerability [3].

Currently, interventional techniques to treat atherosclerotic plaque progression are targeted at increasing and maintaining arterial luminal area by balloon angioplasty, either with or without stent implantation [4]. However, techniques that intervene in the pathophysiological chain of plaque growth and destabilization are scarce [5]. Heat-generating radiofrequency ablation (RFA) has been used to target adventitial sympathetic nerves in treatment-resistant hypertension and has been proven to be safe [6–8]. Like cryotherapy in atherosclerotic vessel disease [9], RFA is known to induce local decellularization and fibrosis formation in healthy arteries [10, 11] and could therefore increase plaque stability. Moreover, similar to adventitial nerve degeneration, we hypothesized that this technique may safely be used to reduce vasa vasorum density and subsequent plaque neovascularization, leading to additional plaque stabilization and possibly decreasing plaque size.

## Methods

### Experimental Design

**Fig. 1:**
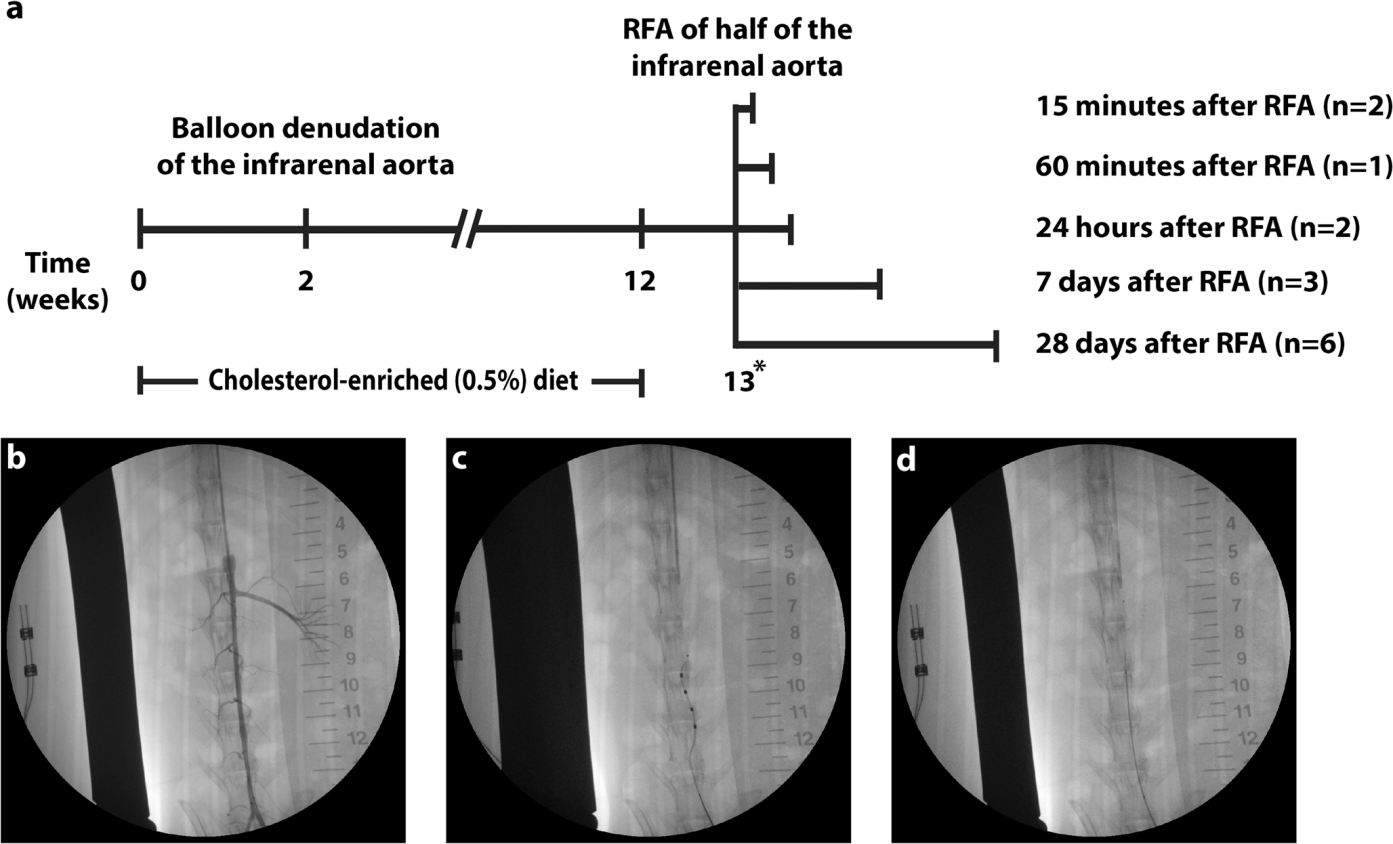
Experimental set-up. Rabbits were fed a 0.5%-enriched cholesterol diet two prior to balloon denudation of the infrarenal aorta and 11 weeks thereafter. One week after cessation of the cholesterol diet, RFA was performed on half of the infrarenal aorta. Rabbits were then sacrificed at different time points (**a**). Prior to RFA, directly after and at termination, angiography of the infrarenal aorta was performed (**b**). In a subset of rabbits, OCT images were acquired (**c**). RFA catheter positioning was established by slightly retracting the guidewire from the lumen of the catheter to let it return to its original spiral shape and assure stable adherence to the vascular wall (**d**). *asterisk:* OCT was performed in a subset of rabbits prior to and directly after RFA

Fourteen male New Zealand White rabbits (Charles River, Chatillon-sur-Chalaronne, R-A, France; 3.0–3.5 kg) were fed a 0.5% cholesterol-enriched diet (Special Diet Services, Essex, UK) for 13 weeks. After 2 weeks, balloon denudation of the infrarenal aorta was performed (Fig. 1a). Male rabbits were used since large-vessel disease is more common in males [12] and to follow up on other atherosclerosis studies that are performed in the same gender [9]. Eleven weeks after balloon denudation, half of the atherosclerotic aorta was treated with transluminal RFA. The other half served as an intra-individual control. Tissue was harvested at different time points after RFA. All animal experiments were approved by the Ethical Committee on Animal Experimentation of the University Medical Center Utrecht (Utrecht, the Netherlands) and conform to the “Guide for the care and use of laboratory animals”.

### Balloon Denudation

Rabbits were fasted overnight before surgery. As premedication, ketamine (15 mg/kg) and xylazine (1.5 mg/kg) were injected intramuscularly. The right inguinal region was shaven and the ear vein cannulated. Subcutaneous meloxicam (1 mg/kg) was given before surgery as analgesia. Directly prior to and every 15–20 min thereafter, a 0.5–1.0-mL mixture of ketamine (10 mg/mL) and xylazine (1 mg/mL) was administered intravenously. Povidone-iodium 2% was applied to the right inguinal region and all other parts were covered with sterile sheets. A small incision was made in the direction of the right femoral artery. The fascia and muscles were bluntly separated and heparin (150 IU/kg IV) was injected prior to cannulation of the right femoral artery. A 4F introducer sheath (Terumo, Leuven, Belgium) was inserted and fluoroscopy was used to advance a 3F Fogarty balloon catheter (Edwards Life Sciences, Irvine, CA, USA) in the abdominal aorta. After inflation, the balloon was retracted through the infrarenal aorta three times to induce endothelial denudation. The Fogarty catheter and sheath were removed from the animal, the wound was closed and the rabbit was allowed to recover.

### Radiofrequency Ablation

Radiofrequency ablation was performed 11 weeks after balloon denudation. Acepromazine and methadone (both 1.5 mg/kg) were injected intramuscularly for premedication. Etomidate (1.5–2 mg/kg) was injected via the ear vein, after which rabbits were intubated and ventilated with a mixture of oxygen/air (1:2) and 1.5% isoflurane. Sufentanil (1 μg/kg/h) was continuously administered intravenously. Subcutaneous meloxicam (1 mg/kg) was given before surgery as analgesia. The right abdominal site was shaven to connect the rabbit to a grounding patch. The neck and inguinal region were then shaven, povidon-iodine 2% was applied, and all remaining parts were draped with sterile sheets. Heparin (150 IU/kg IV) was injected prior to cannulation of the left carotid and femoral artery. A 5F and a 4F sheath were inserted in the respective artery. An angiogram of the infrarenal aorta was made by injecting contrast agent through the lumen of an inflated 4F Fogarty balloon catheter (Fig. 1b). The balloon was deflated and the Symplicity Spyral Radiofrequency Ablation Catheter (Medtronic, Minneapolis, MN, USA) was advanced from the femoral sheath over a 0.014″ Hi-torque Extra S’port guidewire (Abbott Vascular, Libertyville Township, IL, USA) under fluoroscopy. Either the upper or lower half of the infrarenal aorta was randomly selected for treatment with RFA. By slightly retracting the guidewire from the lumen of the RFA catheter, the tip with the electrodes turned to its spiral shape and was firmly positioned to the vascular wall (Fig. 1c). Fluoroscopy images were captured in order to determine the boundary between treated and untreated regions for histological processing. The four electrodes on the device were separately and subsequently activated to perform radiofrequency ablation (≈70 °C) for 2 min under continuous room temperature saline infusion. This was repeated in the adjacent region so that in total 8 points on the aortic wall (half of the infrarenal aorta) were ablated. Afterwards, a control angiogram was performed, the device and sheath were removed from the animal, and the wound was closed. The animal was then allowed to recover from surgery. From the day before RFA until termination, rabbits received 10 mg/kg aspirin (Aspro, Bayer, Mijdrecht, the Netherlands) daily, dissolved in 400 mL freshly prepared drinking water.

### Optical Coherence Tomography

Optical coherence tomography (OCT) was performed in 6 of the 14 rabbits directly prior to, after RFA and at the end of the follow-up period. For this purpose, a C7 Dragonfly™ Duo imaging catheter (St. Jude Medical, St. Paul, MN, USA) was advanced through the femoral sheath and positioned in the infrarenal aorta (Fig. 1d). In order to temporarily remove signal distorting blood flow from the aorta, contrast agent was injected through the lumen of an inflated 4F Fogarty balloon catheter and a manually triggered pullback was performed using the OCT ILUMIEN™ OPTIS™ OCT-system (St. Jude Medical). The CURAD Vessel Analysis program (Curad B.V., Amsterdam, NH, the Netherlands) [13] was used for the assessment of lumen and vessel wall contours in every frame (*n* = 10/mm). Fluoroscopy images with the OCT catheter in situ and matched vessel wall contours were used to specify the control and RFA-treated regions at index procedure and follow-up. Vascular remodeling was assessed by luminal area ratio, resulting from dividing the luminal area at 28 day follow-up by the luminal area directly prior to intervention. The fibrous cap was defined as the hyperintense signal ranging from the luminal border to the inner border of the lower-intensity lipid pool [14].

### Tissue Harvesting

Rabbits were sacrificed 15 min (*n* = 2), 60 min (*n* = 1), 24 h (*n* = 2), 7 days (*n* = 3), or 28 days (*n* = 6) after RFA. Similar anesthetic and surgical protocols as during the RFA procedure were used. Heparin (150 IU/kg IV) was injected prior to cannulation of the right carotid artery. An angiogram was performed to visualize the infrarenal aorta. Subsequently, the abdomen was incised. After heparinization (1000 IU/kg IV), catheters were placed in the aorta and caval vein. After sacrifice, the aorta was perfused with 0.9% saline, followed by pressure fixation with 4% formaldehyde. Subsequently, X-ray was used to determine and mark the boundary between treated and untreated regions as could be retrieved from the RFA catheter positioning images. The aorta was amply explanted from the animal and stored in formalin for at least 24 h. In the rabbit that was sacrificed 1 h after RFA, the aorta was marked, explanted, snap-frozen in liquid nitrogen, and stored at −80 °C.

### Tissue Preparation and Histological Analysis

The treated and untreated region of the infrarenal aorta were each cut axially in 6–8 similar-sized parts and stored at −80 °C (*n* = 1) or embedded in paraffin (*n* = 13). Sections of 4 μm were stained with hematoxylin and eosin (H&E), Elastica van Giesson (EvG), and picrosirius red. In addition, terminal deoxynucleotidyl transferase (TdT) dUTP nick end labeling (TUNEL assay) was performed and immunostains for CD31, alpha-SMA (αSMA), and macrophages.

Cell nuclei in the plaque were stained for H&E and semi-automatically counted using digital histology. EvG staining was used for morphometric analysis. Luminal contours, internal elastic laminae (IEL), and external elastic laminae (EEL) were traced manually using digital histology. Plaque area was calculated by subtracting the luminal area from the IEL area and medial area by substracting the IEL area from the EEL area. Collagen content was quantified in tissue sections stained for picrosirius red and photographed under polarized light. Masson’s trichrome staining was used to identify and measure the fibrotic cap area, which was divided by the luminal perimeter to obtain the average cap thickness per section. Cap thickness was defined as the minimum distance from the luminal border to the inner border of the lipid pool [14].

TUNEL assay was used to identify double-stranded DNA breaks and performed using a DeadEnd™ Colorimetric TUNEL System (Promega, Madison, WI, USA). Of each tissue section, three random fields of the atherosclerotic plaque were selected at ×20 magnification. Nuclei of cells were manually counted and those staining positive for TUNEL expressed as a percentage of the total nuclei in the image. Microvessels in the plaque and adventitia were visualized using a monoclonal mouse anti-CD31 antibody (Clone JC70a, dilution 1:50; Dako). BrightVision poly-AP anti-mouse IgG (Immunologic, Duiven, the Netherlands) was used as a secondary antibody and liquid permanent red (Dako, Glostrup, Denmark) as an enzyme substrate. Only vessels with a lumen smaller than 50 μm in diameter were included in the analysis. Vessel count was corrected for the particular area, with the adventitial area included for analysis extending maximally 200 μm from the EEL. αSMA was used to stain smooth muscle cells. Macrophages were stained using a monoclonal mouse anti-rabbit macrophage antibody (clone RAM-11, dilution 1:800; DAKO). BrightVision poly-AP anti-mouse IgG (Immunologic) was used as a secondary antibody and liquid permanent red (Dako) as an enzyme substrate. Collagen, αSMA and macrophage content are expressed as a percentage of the region of interest (i.e., plaque, media). Images of tissue sections were captured and analyzed using CellSens (Olympus Corporation, Tokyo, Japan).

### Safety

In both, OCT and histology, we evaluated treated and non-treated regions for dissections, thrombus formation, and intraplaque hemorrhage. Dissection was defined as a tear in the wall of the blood vessel that allowed blood to separate the layers. Irregular endoluminal or mural mass on OCT was regarded as thrombus formation. Intraplaque hemorrhage was defined as a fibrin- and/or erythrocyte-rich deposition in the plaque. In addition, attention was paid to clinical symptoms (i.e., hindleg problems).

### Statistical Analysis

In each animal, the individual scores of the tissue sections were averaged per region (i.e., treated vs. untreated, on average eight slides/region). The averages of the treated and untreated regions of all rabbits were then used to calculate the means and standard deviations. Data distribution was evaluated for normality using the Shapiro-Wilk test. Normally distributed data were compared using a paired-samples *t* test; non-normally distributed data were compared using a paired-sample Wilcoxon signed rank test to test for significant differences (*P* < 0.05). Statistical analyses were performed using SPSS software, version 21.

## Results

### Safety Assessment by Angiography, Optical Coherence Tomography, and Histology

**Fig. 2:**
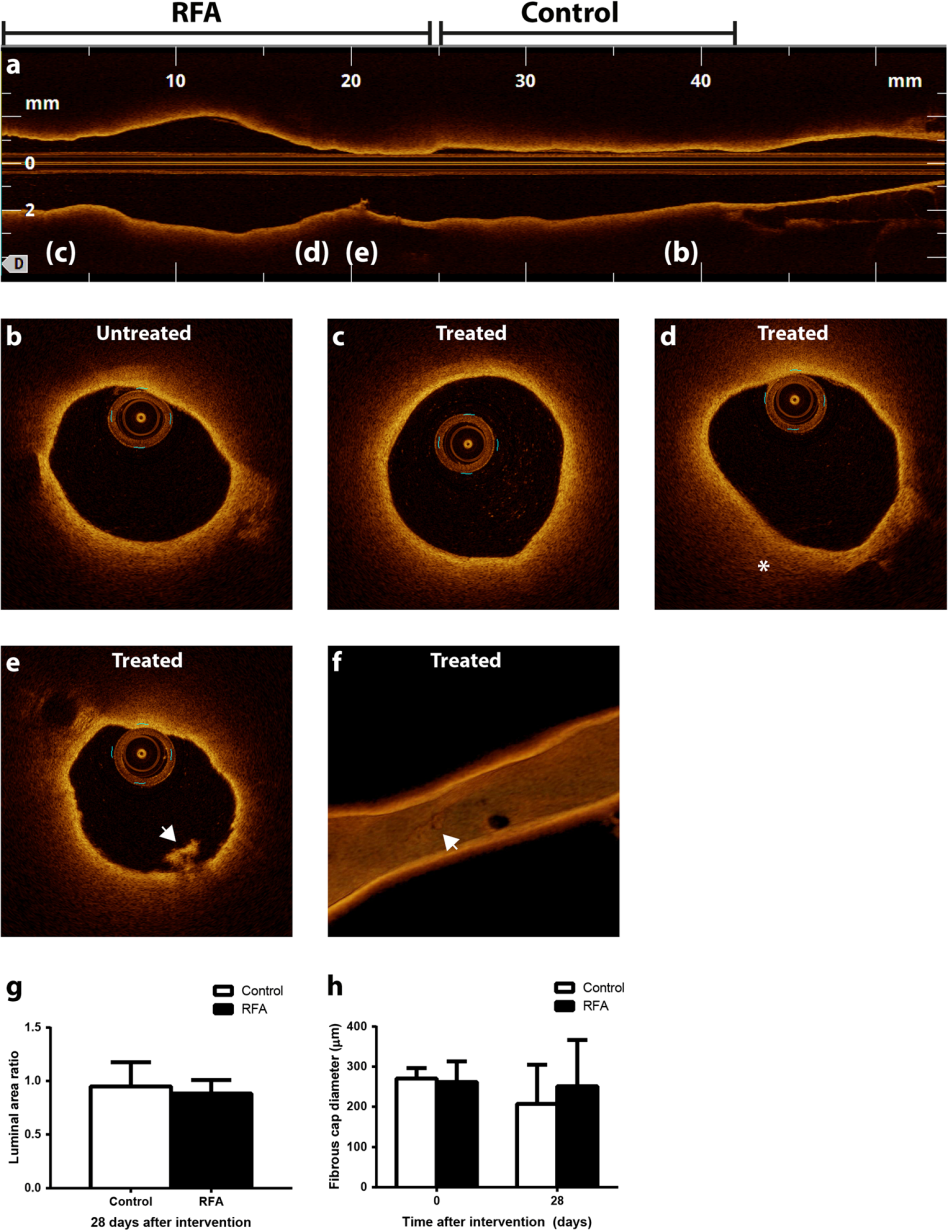
OCT image acquisition. OCT was performed in RFA-treated and control regions of the infrarenal aorta (**a**). The *letters* in this longitudinal image indicate respective transverse images (**b**–**e**). In the majority of the acquired images, control (**b**) and ablated regions (**c**) did not appear to be different. Only incidentally could we observe a slight decrease in signal intensity (*asterisk*) in the RFA-treated area (**d**). In one rabbit in one location, we observed thrombus formation without clear dissection (**e**), with a 3D reconstruction showing the extent of the thrombus formation in the vessel wall (*arrow*) (*f*). Luminal area ratio of area at 28-day follow-up divided by area prior to RFA showed no difference in vascular remodeling between control and remote regions (*g*), nor did fibrouw cap thickness differ at baseline or follow-up (**h**)

RFA did not induce any macroscopic effects detectable with angiography. We did not observe vasospasms or oedema at the site of RFA, nor did we detect any adverse events such as dissection or thrombus formation. To increase sensitivity, we performed OCT prior to and directly after RFA in six rabbits (Fig. 2a). Again, we could not detect any vessel wall oedema, plaque rupture, or dissection. Overall, the vessel wall in RFA-treated regions was not distinguishably different from control regions (Fig. 2b, c). Directly after RFA, we observed a decrease in signal intensity in one rabbit (out of six) (Fig. 2d). In one rabbit, we detected a small thrombus without evident vessel wall narrowing or dissection (Fig. 2e, f). Luminal area ratio showed comparable remodeling in both regions (0.95 ± 0.23 vs. 0.88 ± 0.13; *p* = 0.77, Fig. 2g). In addition, cap thickness did not differ between control and RFA-treated regions prior to (271 ± 26 vs. 262 ± 51 μm; *p* = 0.65, Fig. 2h) and 28 days after intervention (207 ± 98 vs. 251 ± 115 μm; *p* = 0.08).

**Fig. 3:**
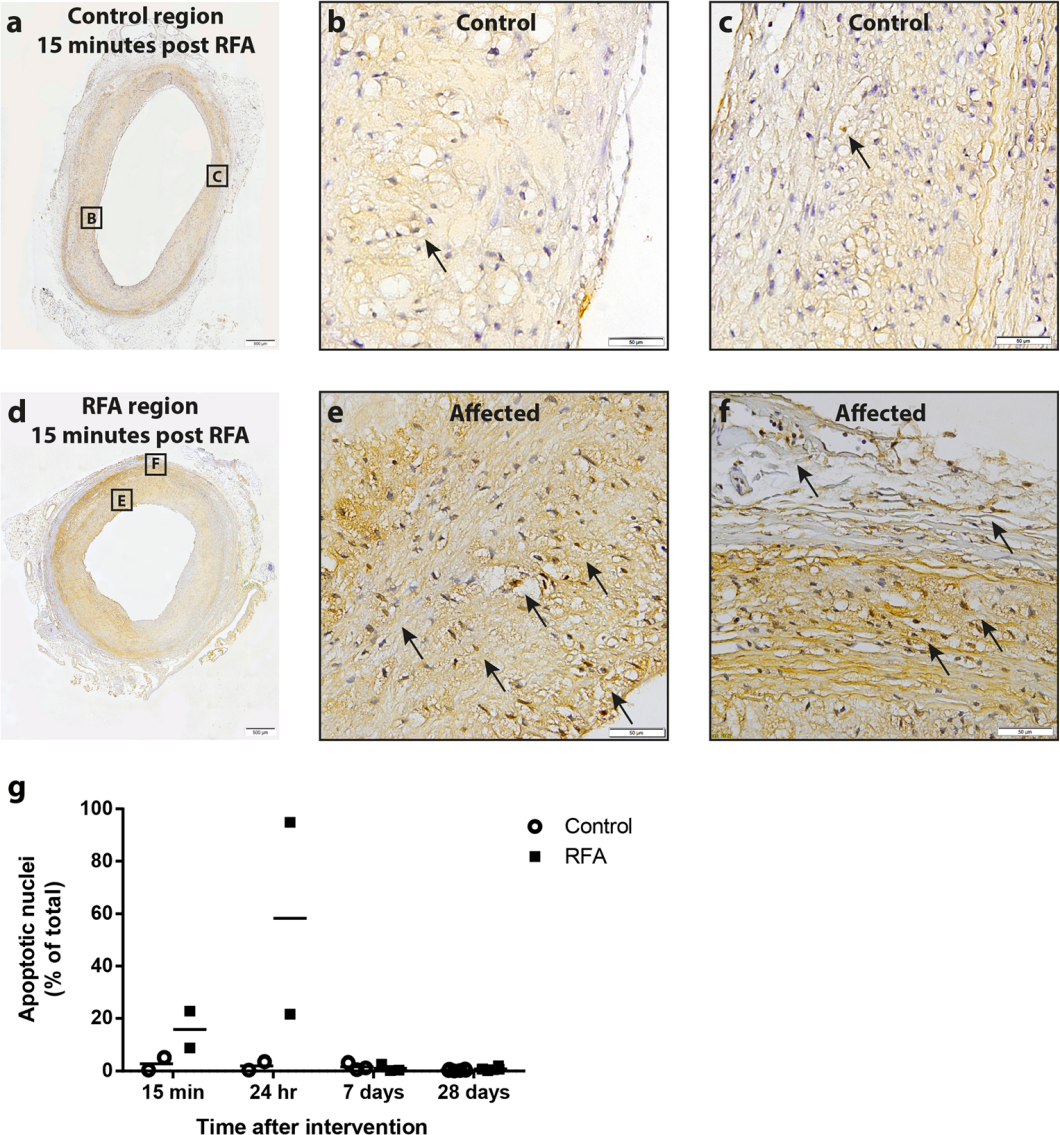
DNA damage in RFA and control regions. TUNEL assay was performed to detect double-stranded DNA breaks as a marker for apoptosis in both control and RFA-treated regions. Control regions showed only few nuclei staining positive (*brown*) (**a**–**c**), whereas in specific RFA-treated regions, a considerable amount of nuclei were shown to stain positive up to the adventitia (**d**–**f**). This effect was most apparent 15 min and 24 h after RFA and was abolished at longer term follow-up (**g**). Positive nuclei are indicated by *arrows*

On histological examination no evidence was found for dissection, thrombus formation, or other adverse events in any of the rabbits. TUNEL assay was used to localize focal ablation points and to allow for identification of blunt ends of double-stranded DNA breaks. Untreated regions showed few to no nuclei staining positive (brown) for TUNEL (Fig. 3a–c). On the contrary, treated regions of rabbits sacrificed within 24 h after RFA showed an increased number of nuclei staining positive for TUNEL, extending from the plaque to the adventitia (Fig. 3d–f), without breaching the endothelium. Compared to untreated control regions, the percentage of positive nuclei in the treated region was increased 15 min (2.7 ± 3.3 vs. 15.7 ± 10.0%; *p* = 0.22, Fig. 3g) and 24 h after RFA (2.0 ± 2.2 vs. 58.3 ± 51.8%; *p* = 0.38).

### Lesion Characterization and Cellularity

**Fig. 4:**
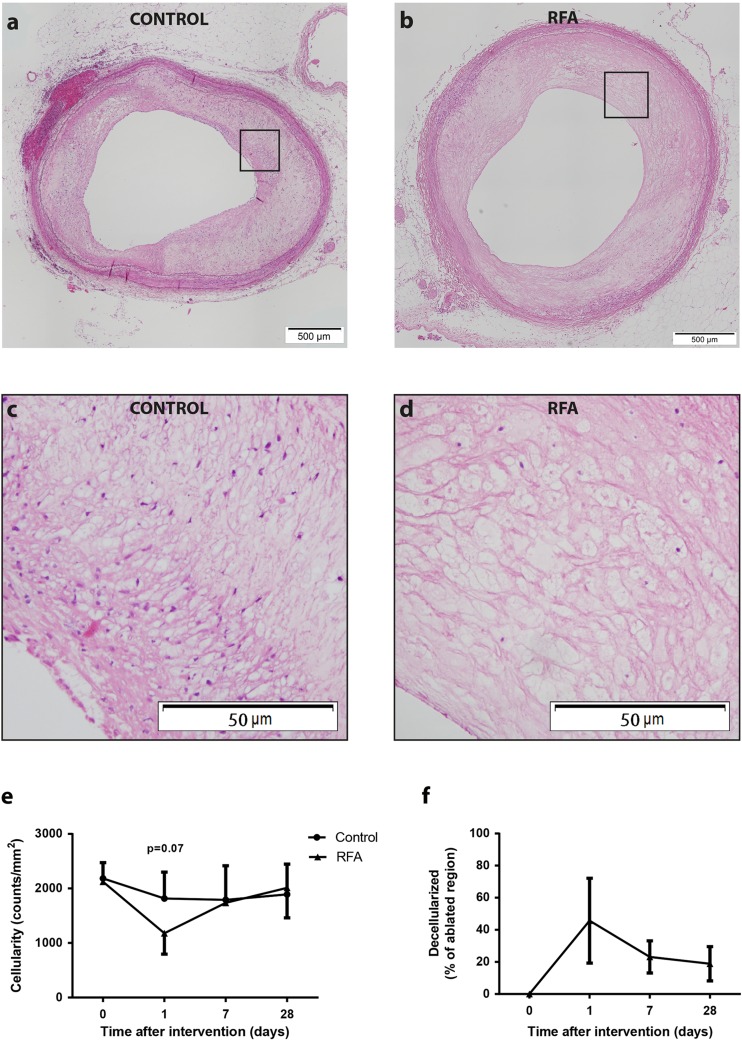
Plaque cellularity and percentage affected region. Representative HE-stained tissue sections of control (**a**) and treated (**b**) regions and respective magnifications (**c**, **d**) showed that RFA greatly reduced cellular content. This effect was most pronounced 24 h post-RFA, after which cellularity was normalized at 7 and 28 days (**e**). The decellularized area was quantified as a percentage of the total region where RFA was performed and showed that the effect of RFA was most apparent after 24 h (**f**)

Tissue sections from RFA-treated areas from rabbits sacrificed 24 h, 7 days, or 28 days after RFA showed lesions characterized by (locally) decreased cellularity (Fig. 4a–d). As a consequence, a trend towards decreased intimal cellularity 24 h after RFA was observed (1817 ± 482 vs. 1180 ± 383 nuclei/mm^2^; *p* = 0.07, Fig. 4e). After 7 or 28 days, ≈20% of the plaque in the treated region remained decellularized and intimal cellularity was no longer different from untreated regions (Fig. 4f).

### Vessel Density

**Fig. 5:**
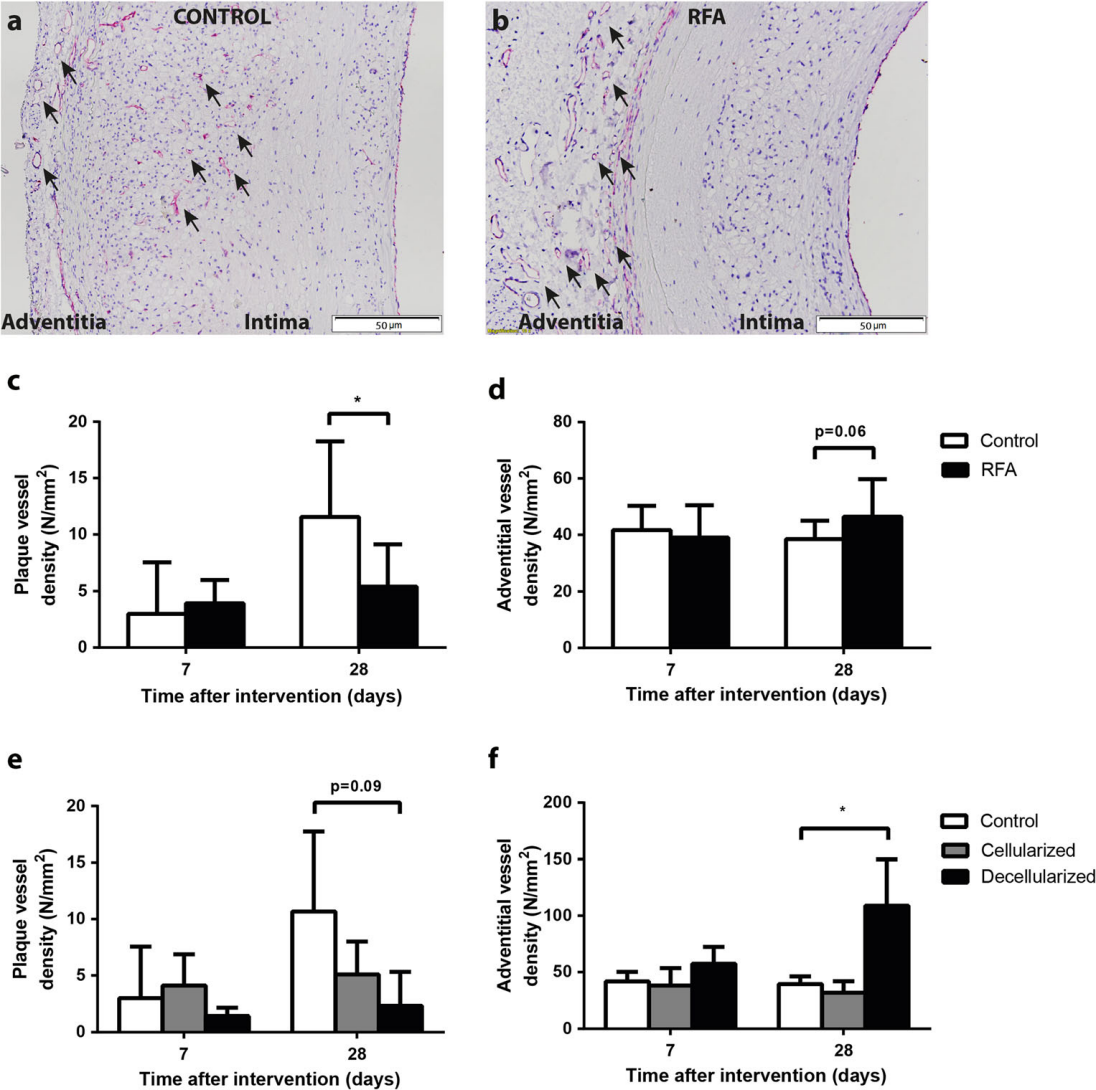
Plaque and adventitial vessel density. Representative images of anti-CD31 staining are shown for control (**a**) and treated plaques (**b**). Vessel density in the plaque at 28 days was lower in regions treated by RFA when compared to control regions (**c**). With respect to adventitial vessel density, no significant differences were observed between both groups (**d**). Further exploration of cellularized and decellularized areas showed that the difference between treated and untreated regions after 28 days was mainly explained by a decrease in plaque vessel density in the decellularized area (**e**). With respect to adventitial density, the increase in treated regions after 28 days could be mainly attributed to an increase in decellularized areas (**f**). Plaque vessels and vasa vasorum are indicated by black arrowheads. *p < 0.05

Figure 5a, b shows CD31 staining in both the plaque and adventitia. Plaque vessel density was not different between untreated and treated regions 7 days after RFA (3.0 ± 4.6 vs. 3.9 ± 2.1 vessels/mm^2^; *p* = 0.76, Fig. 5c). At 28 days, vessel density in the plaque was significantly lower in RFA-treated regions (11.6 ± 6.7 vs. 5.4 ± 3.7 vessels/mm^2^; *p* = 0.028). With respect to adventitial vessel density, no significant differences were observed 7 days (41.7 ± 8.6 vs. 39.1 ± 11.5 vessels/mm^2^; *p* = 0.81, Fig. 5d) or 28 days (38.5 ± 6.6 vs. 46.4 ± 13.4 vessels/mm^2^; *p* = 0.06) after RFA.

To further explore whether differences existed in the regions due to a local and non-circumferential effect of RFA, we separately quantified vessel density in both the cellularized and decellularized areas in the treated region. We selected the adventitial area extending up to 200 μm from the EEL and directly adjacent to the decellularized plaque and media. This showed that the difference in plaque vessel density after 28 days could be attributed to a decrease in the part of the plaque that remained decellularized (control 10.7 ± 7.1 vs. decellularized area 2.3 ± 3.0 vessels/mm^2^; *p* = 0.09, Fig. 5e). In contrast, adventitial plaque vessel density was significantly increased in the decellularized region when compared to the control region (control 39.4 ± 6.9 vs. decellularized area 108.9 ± 40.9 vessels/mm^2^; *p* = 0.014, Fig. 5f).

### Atherosclerotic Plaque Burden and Plaque Composition

According to the modified AHA Classification, the great majority of the plaques in the current study most closely resembles the fibroatheromatous plaque type without the presence of a necrotic core or calcifications [15].

**Fig. 6:**
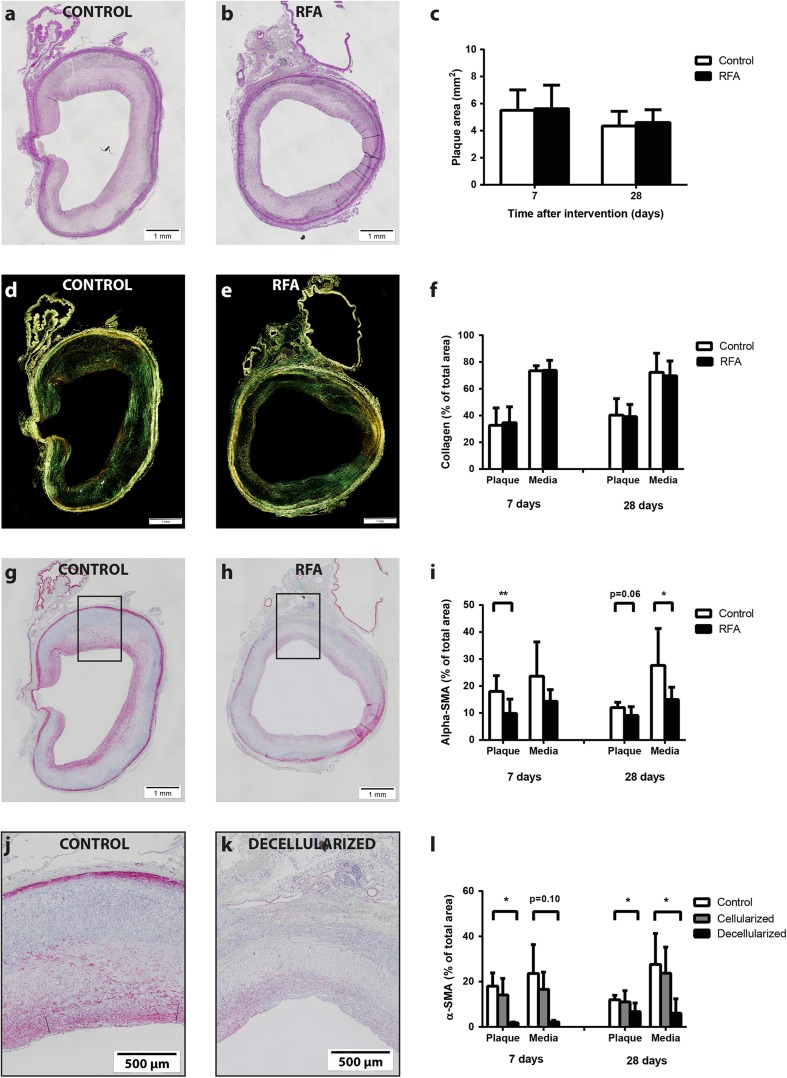

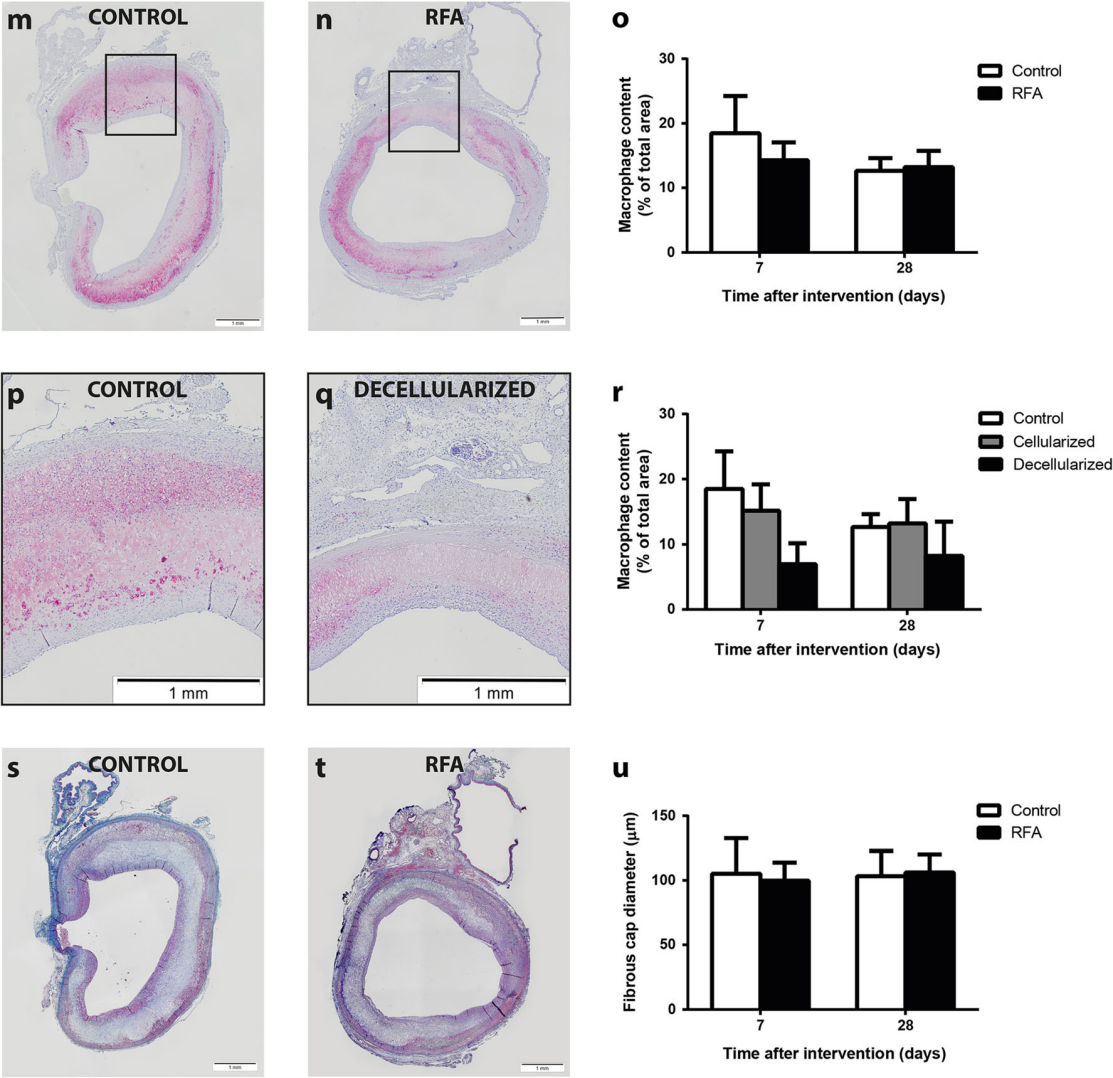
Atherosclerotic plaque burden and plaque composition. An EvG staining was used to quantify plaque burden in control (**a**) and RFA regions (**b**). Both, after 7 and 28 days, no difference was observed with respect to atherosclerotic plaque area (**c**). Collagen content was quantified by picrosirius red staining (**d**, **e**) and did not show any differences between either the plaque or media of both regions (**f**). Smooth muscle cell content assessed by αSMA staining (**g**, **h**) showed a major decrease in the plaque and media of regions treated with RFA at either 7 or 28 days (**i**). Magnified images hereof (**j**, **k**) show that this effect could mainly be attributed to areas that remained decellarized after RFA treatment (**l**). Macrophage content in control (**m**) and RFA (**n**) regions was not different (**o**). Although not significant, decellularized areas showed a trend towards lower macrophage content compared to control regions, mainly after 7 days (**p**–**r**). Cap thickness assessed by masson’s trichrome staining of control (**s**) and RFA (**t**) regions was not different between both regions (**u**). *p < 0.05, **p < 0.01

Plaque burden by EvG staining (Fig. 6a, b) did not differ between untreated and treated regions at 7 days (5.5 ± 1.5 vs. 5.6 ± 1.7 mm^2^; *p* = 0.62, Fig. 6c) or 28 days (4.3 ± 1.1 vs. 4.6 ± 0.9 mm^2^; *p* = 0.46) after RFA. In addition to serial OCT measurements providing luminal area ratio, we also used IEL area at 28-day follow-up as a measure of remodeling. [16, 17] Although RFA-treated regions seemed to have slightly more outward remodeling, this difference was not significant (8.7 ± 1.2 vs. 10.3 ± 2.6; *p* = 0.07). Collagen content in the plaque as assessed by picrosirius red staining was comparable between both groups at 7 days (32.7 ± 13.0 vs. 34.6 ± 11.9%; *p* = 0.22, Fig. 6d–f) and 28 days (40.3 ± 12.4 vs. 39.2 ± 9.1%; *p* = 0.85) and similarly in the media at 7 days (73.4 ± 3.8 vs. 73.8 ± 7.5%; *p* = 0.89) and 28 days (72.3 ± 14.3 vs. 69.7 ± 11.1%; *p* = 0.72). Representative examples of αSMA stains are displayed in Fig. 6g, h. Compared to untreated regions, αSMA content as a percentage of the plaque area was significantly decreased in treated regions at 7 days (18.0 ± 5.9 vs. 9.8 ± 5.3%; *p* = 0.006, Fig. 6i) and a trend was observed at 28 days after RFA (12.0 ± 2.0 vs. 9.1 ± 3.3%; *p* = 0.06). Moreover, medial αSMA content was decreased 28 days after RFA (27.7 ± 13.6 vs. 15.0 ± 4.6%; *p* = 0.033). Similar to vessel density quantification, we separately quantified αSMA content in both the cellularized and decellularized areas in the treated region. This pointed out that differences between control and treated regions could mainly be explained by decreased αSMA content in the decellularized part of the treated region after 7 days (control 18.0 ± 5.9 vs. decellularized 1.8 ± 0.4%; *p* = 0.038, Fig. 6j–l) and 28 days (12.6 ± 1.6 vs. 6.8 ± 3.8%; *p* = 0.020). Similarly, the media of treated decellularized regions was lower in αSMA content after 7 days (23.6 ± 12.7 vs. 2.2 ± 0.7%; *p* = 0.10) and 28 days (32.0 ± 9.7 vs. 6.1 ± 6.4%; *p* = 0.013).

In addition, macrophage area as a percentage of the plaque did not differ between untreated and treated regions at 7 days (18.5 ± 5.8 vs. 14.3 ± 2.8 mm^2^; *p* = 0.32, Fig. 6n- o) and 28 days (12.6 ± 2.0 vs. 13.2 ± 2.5%; *p* = 0.73). Although not significant, subanalyses indicated that decellularized parts of the treated area were lower in macrophage content than control regions after 7 days (control 18.5 ± 5.8 vs. decellularized area 7.0 ± 3.2%; *p* = 0.11, Fig. 6p–r). This difference was not observed after 28 days, when macrophage content was comparable between both areas (12.6 ± 2.0 vs. 8.2 ± 5.2%; *p* = 0.17).

Representative images of cap thickness in both control and RFA-treated regions are shown in Fig. 6s, t. No difference was observed between both regions 7 days (105 ± 27 vs. 100 ± 14 μm; *p* = 0.71, Fig. 6u) and 28 days after RFA (103 ± 20 vs. 106 ± 14 μm; *p* = 0.72). Similarly, no differences in subanalyses of control, decellularized, or cellularized regions were observed after 7 days (control 105 ± 27 vs. decellularized 109 ± 29 μm; *p* = 0.39) and 28 days (control 101 ± 21 vs. decellularized 103 ± 24 μm; *p* = 0.90).

## Discussion and Limitations

To our knowledge, this is the first report on radiofrequency ablation (RFA) of atherosclerotic lesions. RFA is safe and modulates plaque vessel density and smooth muscle cell content in a rabbit atherosclerosis model.

### Safety

Other than a small thrombus without vessel narrowing in one of the animals after RFA as evidenced by OCT, we did not observe any adverse events after treatment. RFA induced thrombus formation is frequently observed by OCT in preclinical and clinical renal denervation studies without adverse angiographic or clinical relevance [6–8, 10, 18], whereas this was an incidental finding in our study. We performed RFA under continuous saline irrigation, possibly preventing blood clotting by lowering blood temperature. In addition, coagulation in pig and human blood may differ from rabbit blood [19] and the heparin dose of 150 IU/kg given is considerably higher than normally administered to patients.

Apoptosis examination by TUNEL assay showed RFA induced double-stranded DNA breaks in all vascular layers without endothelial disruption. The current application of RFA exerted a rather local effect, reflected by persistent decellularization. This corresponds to the tissue ablation and cell depletion observed in previous studies performing RFA on the arterial vessel wall [10, 11].

### Vessel Density

Although the TUNEL assay confirmed that thermal energy indeed reached the adventitia, no significant decrease in vasa vasorum density was observed. In fact, adventitial vessel density was even higher in decellularized regions closest to the applied radiofrequent energy. This could be due to insufficient capillary degradation or a reactive angiogenic response in the adventitia after RFA, as described previously [10]. In contrast, plaque vascularization was significantly decreased in RFA-treated regions 28 days after RFA. Leaky neovessels are the main origin of intraplaque hemorrhage leading to vulnerable plaque phenotypes and worse clinical outcome [20]. Thus, RFA might serve as a means to decrease intraplaque hemorrhage in more advanced plaques. The discrepancy between adventitial and plaque (neo)vascularization may suggest that only more severe tissue ablation results in decellularization, thereby creating an environment that is less susceptible to vascular penetration. This finding is supported by the observation that—although the majority of the plaque was recellularized after RFA—local areas remained decellularized up to 28 days thereafter and showed lower vessel density than recellularized areas in the treated region.

### Plaque Composition

Although RFA has been shown to increase collagen content and induce fibrosis in healthy arteries [10], this was not observed in the present study. This may be due to the already relatively high collagen plaque content in this model. The unchanged collagen content may also explain the lack of difference in remodeling in both regions, since changes in the extracellular matrix are of key importance in the process of arterial remodeling [16].

In contrast, RFA-treated regions showed a trend towards reduced overall cellularity after 24 h and macrophage content up to 7 days. After 28 days, no differences between both regions were observed. Most likely, cell death resulted in a secondary low grade inflammatory reaction to clear the plaque from debris [11], culminating in comparable cellularity and macrophage content at long-term follow-up. Surprisingly however, RFA did not evoke a disproportionate inflammatory response, since macrophage content did not differ between RFA-treated and control regions at 7 and 28-day follow-up. This finding is in agreement with the considerable proportion of TUNEL positive nuclei, indicating apoptotic cell death [21, 22]. These findings suggest that apart from the normally observed thermal coagulation necrosis [23], apoptosis is at least to some extent responsible for cell death in the present study.

In addition, cap thickness was not affected by RFA. Although both methods show comparable cap thickness between the regions, the difference in absolute values can be explained by the observation that—in optical coherence tomography—high-fat plaques have irregular and not well-delineated borders [24], as opposed to human plaques [14]. This increases scattering on OCT images, decreasing the feasibility to clearly delineate the fibrous cap from the inner border of the lipid pool.

Importantly, although RFA treatment did not influence cap thickness, SMC content was decreased 7 and 28 days after RFA in both intima and media. While decreased SMC content could potentially compromise plaque stability [2] and therefore needs to be carefully evaluated, it may hint at a potential benefit in preventing (re)stenosis in peripheral arterial disease (PAD) and coronary artery disease (CAD), where SMC proliferation is a key mechanism. Despite the latest advances in stent design, restenosis rates remain relevant with 10–20% at 12-month follow-up for coronary artery stents and significantly higher rates for peripheral stenting [5]. Since plaque burden is not influenced by the current technique, further studies combining simultaneous radiofrequency energy delivery and balloon angioplasty could offer improved results.

Although the current study provides important insight in the effect of RFA on atherosclerotic plaques, we would like to discuss some of its limitations. Due to the limited numbers—especially up to 24 h after RFA—we were not able to compare vessel density in the subacute phase. Moreover, although serial detection of neovessels using OCT has been described [25], capillaries were rather large (50–300 μm) as opposed to the rabbit neovessels in the current manuscript (~10 μm), hampering their reliable detection. In addition, atherosclerotic animal models, including the present, do not develop advanced atherosclerotic lesions, which limits translation to the human situation. Although it may be interesting to evaluate the effect on a more advanced plaque phenotype, the rabbit model offers us a good alternative approach to appreciate the effects on atherosclerotic plaques. Moreover, a longer follow-up period could be informative to show whether plaque composition (i.e., smooth muscle cell content) would eventually normalize and if plaque burden would be affected (i.e., would decrease). In this regard, serial morphometric measurements could provide insight into plaque burden over time. Finally, the local effect of RFA might limit its ability to influence plaque burden over the total treated region. Therefore, an approach with a more advanced RFA catheter that targets the whole circumference could be of interest.

In conclusion, radiofrequency ablation is safe in moderate atherosclerotic vessel disease. It leads to near-complete plaque decellularization in treated areas in the subacute phase, a decrease in plaque vessel density, and a major reduction in local smooth muscle cell content. Yet, 7 and 28 days after intervention, it does not reduce vasa vasorum or affect plaque volume, cellularity, cap thickness, or collagen content in a rabbit atherosclerotic model. Therefore, combining this technique with balloon angioplasty could be promising in the treatment of severe (re)stenosis in (peripheral) arterial disease.

## Abbreviations

AMI: Acute myocardial infarction
αSMA: Alpha-smooth muscle actin
CVA: Cerebrovascular accident
CVD: Cardiovascular disease
EEL: External elastic laminae
EvG: Elastica van Giesson (EvG)
F: French
H&E: Hematoxylin and eosin
IEL: Internal elastic laminae
IV: Intravenous
OCT: Optical coherence tomography
RFA: Radiofrequency ablation
SMC: Smooth muscle cell
TUNEL: Terminal deoxynucleotidyl transferase (TdT) dUTP nick end labeling
